# Feasibility study of Glucagon-like peptide-1 analogues for the optimization of Outcomes in obese patients undergoing AbLation for Atrial Fibrillation (GOAL-AF) protocol

**DOI:** 10.1186/s40814-024-01454-y

**Published:** 2024-02-21

**Authors:** Kyaw Z. Win, Matthew Armstrong, Richard P. Steeds, Manish Kalla

**Affiliations:** 1 Department of Cardiology, Queen Elizabeth Hospital Birmingham, University Hospitals Birmingham NHS Foundation Trust, Birmingham, UK; 2 Institute of Cardiovascular Sciences, University of Birmingham, Birmingham, UK; 3 Department of Hepatology, Queen Elizabeth Hospital Birmingham, University Hospitals Birmingham NHS Foundation Trust, Birmingham, UK

**Keywords:** Atrial fibrillation, Weight loss, Liraglutide, Feasibility, Phenotyping

## Abstract

**Background:**

Catheter ablation for atrial fibrillation is recommended for symptomatic patients after failed medical therapy. Ablation has a higher failure rate in obese patients, and both the prevalence of atrial fibrillation and obesity are increasingly globally. The outcome of ablation can be improved if obese patients can achieve goal-oriented weight reduction prior to ablation. Conventional weight loss strategies, however, can be difficult to access and can delay ablation, thereby risking a lower chance of maintaining sinus rhythm. Effective weight-loss medications, such as the glucagon-like peptide inhibitor-1 drugs, offer the potential for incremental impact on weight loss over a shorter period of time as a bridging therapy. The aim of this study is to assess the feasibility of using liraglutide, a glucagon-like peptide inhibitor-1, in producing weight loss in obese patients before catheter ablation.

**Methods:**

The study is an open-label, uncontrolled, prospective single-centre feasibility study of daily liraglutide injections in the treatment of obese patients for at least 13 weeks before and 52 weeks after AF ablation. Adult patients with symptomatic AF whose body mass index ≥ 30 will be recruited from those planning to undergo ablation. Feasibility will be determined based on the recruitment rate, adherence to the medication, and the amount of weight loss achieved over the study period. Exploratory outcomes include changes in atrial structure, function, and fibrosis with weight loss evaluated by cardiac magnetic resonance imaging, electroanatomic mapping, and patient-reported outcome measure.

**Discussion:**

This study will allow us to determine whether the use of liraglutide in obese patients with atrial fibrillation undergoing ablation is feasible with adequate recruitment. The additional information on adherence and average weight loss over the study period will inform the design of a future definitive randomized controlled trial.

**Trial registration:**

ClinicalTrials.gov (NCT05221229). Registered on 2 February 2022.

**Trial funding:**

Metchley Park Medical Society and University of Birmingham Starter Fellowship, British Heart Foundation Accelerator Grant, Abbott Investigator-Initiated Study Grant.

**Supplementary Information:**

The online version contains supplementary material available at 10.1186/s40814-024-01454-y.

## Background

Atrial fibrillation (AF) is the most common arrhythmia in clinical practice, and the prevalence is expected to rise worldwide. AF increases the risk of hospital admissions, stroke, dementia, and mortality, and it lowers quality of life (QoL). The prevalence of obesity has tripled since 1975 [1] and is increasingly recognized as a stand-alone risk factor for AF independent of associated conditions such as hypertension, diabetes mellitus, and obstructive sleep apnoea [2–4]. The risk of developing AF [5], likelihood of progression [6], and a poor response to its management [7, 8] are all increased with progressive weight gain. Rhythm control therapies such as direct current cardioversion, antiarrhythmic drugs, and catheter ablation are important treatments of AF in maintaining sinus rhythm, improving symptoms and QoL, and reducing repeated hospitalization [9]. Cather ablation is, however, the most effective rhythm control therapy for symptomatic AF patients unresponsive to medications [10–13]. Accumulating evidence suggests that rhythm control management should be considered as early as within 1 year of AF diagnosis [14–17]. However, obesity (body mass index, BMI ≥ 30) lowers the success rate of ablation by up to 10% [18]. Multiple studies have reported that goal-directed weight reduction and optimization of risk factors can reduce AF progression, improve both single and multi-procedure success rates following ablation, and long-term maintenance of sinus rhythm [19–22].

In clinical practice, conventional weight loss strategies such as intensive lifestyle modification, pharmacotherapy such as orlistat, and bariatric surgery have limitations. These include limited availability across all healthcare systems, poor tolerability, invasive nature, and delay in achieving the required weight loss. As a result, an alternative or bridge therapy for this cohort should be explored. The newer weight loss medications such as liraglutide and semaglutide have potentials to close this gap [23, 24]. In this study, liraglutide was selected in preference to semaglutide due to its long-term safety [25].

The primary outcome for a future definitive randomized controlled trial comparing goal-oriented weight reduction with liraglutide together with risk factor modification group to risk factor modification alone will be the effect on AF-free survival at 52 weeks following AF ablation. Such a study cannot be adequately designed without evidence that liraglutide can achieve weight loss in this cohort between identification of the need for ablation and the procedure (on average 13 weeks), that adherence is maintained before and after ablation, and that recruitment rates are sufficient. This knowledge is essential to design a future definitive trial.

### Aims and objectives

The primary aim of this feasibility study is to determine whether liraglutide injected 3 mg once daily for 13 weeks before first-time AF ablation and continued for 52 weeks after ablation is a feasible method of reducing the recurrence and burden of AF (reported as percentage of total recordings) detected by Kardia portable electrocardiogram (ECG) recordings (at least twice a day and according to symptom-triggered recordings) after catheter ablation in patients with paroxysmal or persistent AF and body mass index above 30. In our centre (Queen Elizabeth Hospital, Birmingham, QEHB), the usual waiting time for patients for their first-time ablation is at least 12 weeks. A shorter waiting time is unusual in the United Kingdom (UK), and a longer waiting time needs to be avoided, as delay increases risk of AF recurrence.

Feasibility (the primary objectives) of the study will be determined by the measurement of the following:

- The number of participants recruited as a proportion of those eligible for the study over the recruitment period
- The proportion of participants adherent to liraglutide until the end of 52 weeks follow-up after ablation
- The percentage of weight loss (from recruitment) achieved at the time of ablation and the end of follow-up period after ablation.

Exploratory outcomes will also be determined as follows:

- The proportion of participants adherent to Kardia recording requirements (at least twice daily recordings over 52 weeks)
- The time-to-first recurrence of AF (> 30 s) beyond 12-week blanking period post ablation
- The burden of AF (percentage of total observed time) beyond 12-week blanking period post ablation in predefined periods (13th–26th weeks, 27th–39th weeks, and 40th–52nd weeks following ablation)
- Changes in cardiac MRI (CMR) measures from baseline to before ablation
  - ◦ Epicardial adiposity (volume measure in cm^3^)
  - ◦ Left atrial fibrosis (percentage of scar to total atrial volume rendered in CEMERGAPP by using late gadolinium-enhanced left atrial CMR images)
- Changes in fibroblast growth factor (FGF)-23 (pg/ml) and bone morphogenic protein (BMP)-10 (ng/ml) levels from baseline to before ablation
- Changes in electro-anatomic mapping data (right atrial bipolar voltage [mV] and conduction velocity [cm/s]), from baseline to before ablation

## Methods and design

### Study design and population

This is an open-label, uncontrolled, prospective, single-centre feasibility study of liraglutide in the treatment of obese AF patients injected for at least 13 weeks before and 52 weeks after AF ablation. Patients waiting for first-time AF ablation in QEHB will be invited for the study. We aim to recruit 30 patients who meet the eligibility criteria. Potential participants will be screened through cardiology outpatient clinics and the waiting list for AF ablation at QEHB.

#### Inclusion criteria

- Adult patients with paroxysmal or persistent AF (> 18 years of age)
- Symptomatic: Defined as palpitations, shortness of breath, and feeling of an irregular pulse or pause in heart activity, despite guideline-directed medical therapy (or inability to take medications)
- Body mass index above 30 kg/m^2^ (Mosteller equation)

#### Exclusion criteria

- A contra-indication or caution to treatment with liraglutide or to any study procedure, including CMR
- Previous or current use of medication or planned surgery for weight loss
- Type 1 diabetes
- Type 2 diabetes on DPP-IV inhibitor or insulin

### Study setting, patient identification, and recruitment

QEHB is the main tertiary referral centre for AF ablation in Birmingham, the second largest city in the UK with a population of 1.1 million [26]. All the research will take place in QEHB. The study is supported by the cardiology research group and electrophysiology team. No additional screening, including laboratory and diagnostic testing, is required to meet the inclusion and exclusion criteria of the study. Eligible participants will be screened through cardiology outpatients’ clinics and the waiting list for AF ablation run by the electrophysiology consultants. Identified patients will be approached by members of the clinical team and, if interested, will be given a patient information sheet (PIS) to facilitate the consenting process. All participants who are eligible into the study will then go through a formal consent process with one of the research team members, according to good clinical practice guidelines. During the process, adequate time will be given to the potential participants to decide whether to take part in the study. Patients who are currently participating in other research projects will not be recruited.

### Justification of sample size

Feasibility studies are not designed to prove the superiority of one treatment over another. Instead, the aim is to test whether the proposed intervention is a feasible method of treatment in this group of patients. Our internal unpublished data suggests QEHB performs first-time AF ablation on approximately 140 patients per calendar year, and 40% (56) of patients will be eligible for inclusion. We aim to recruit 30 patients in total by assuming just over 50% of eligible patients will agree to participate with an approx. 10% dropout rate. We expect 70% (21) of recruited participants to remain adherent to liraglutide at the end of the study based on previous published data [23]. Using the binomial exact method for this proportion results in the 95% confidence interval being 15–25 recruited participants to remain adherent to liraglutide at 52 weeks following ablation. As there is no current study recruiting similar patients, the investigators expect to complete recruitment within 52 weeks.

### Intervention

As per British National Formulary, liraglutide will be injected 0.6 mg once daily, increased in steps of 1 week up to a maintenance dose of 3 mg once daily, or the maximally tolerated. If still clinically indicated at the end of study, liraglutide will be continued and followed up as per the National Health Service (NHS) standard care. This will be reported as cumulative doses of liraglutide over the study period, considering any disruption due to potential side effects. Adherence will be checked during monthly telephone follow-ups and return of empty pens via pre-paid envelopes from the participants.

### Other aspects of care

Consistent with current standard of care, all participants will be offered lifestyle advice, including on weight reduction, monitoring of hypertension, advice regarding diabetic optimization, and reduction in alcohol.

### Feasibility outcome measures

The study will be considered feasible if as follows:

- Recruitment rate ≥ 50% (≥ 30 patients) of those eligible (56 patients per year), drop-out rate < 10%, and data completeness ≥ 90%
- Adherence to medication rate ≥ 70% in the participants at the end of study [23]. Adherence will be measured by the proportion of patients who tolerate total cumulative dose of 657 mg and more of liraglutide at 1 year (minimal maintenance dose of 1.8 mg over 365 days).
- Weigh loss at least 5% from baseline in ≥ 60% participants at the end of study [23, 27]. Body mass change in kilograms and BMI between the baseline, at ablation, and last days of treatment with liraglutide (± 7 days) will be measured using a single set of calibrated scales using a standardized protocol.

### Secondary outcome measures

#### Recurrence of AF

Recurrence of AF is defined as a heart rhythm with no discernible p waves and irregular RR intervals, when atrioventricular conduction is not impaired on a standard 12 lead ECG or a recording of a single-lead ECG tracing of equal or more than 30 s, by Kardia recording. Recurrence will be determined time-to-first recurrence after a 12-week blanking period (> 30-s AF, secondary end point).

#### AF burden

AF burden will be quantified by twice daily and symptom-triggered (onset and termination) Kardia recordings which will be sent daily. The time of the recorded AF event will be taken as the onset of the episode, with the next recorded sinus rhythm taken as the offset.

#### Changes in CMR

CMR will be performed by using 1.5-T scanner (MAGNETOM Verio, Siemens, Erlangen, Germany) of QEHB. ECG R-wave-gated, steady-state-free precession imaging (SSFP) [True-FISP] cine images of the left and right ventricle in vertical long axis, horizontal long axis, and serial contiguous short axis will be acquired, throughout the atria and ventricles according to standard parameters. Epicardial adiposity (EpAT) will be measured from atrioventricular (AV) grove to apex during ventricular end systole in short-axis stack images by contouring of each slide. The total volume will then be calculated by adding each slide. Late gadolinium images of the left atrium will be taken 15 min following intravenous gadolinium administration (0.1 mmol/kg). These images will be used to render scar images in an open-sourced CEMRGAPP to calculate left atrial fibrosis. The method of rendering has been published by CEMRGAPP group elsewhere [28]. Both clinical and research scans will be reported by an experienced imaging cardiologist (RPS).

#### Biomarkers

Blood samples will be collected in QEHB at baseline and before ablation. The samples will be transported to the Institute of Cardiovascular Sciences at the University of Birmingham at room temperature for testing and storage. Samples will be stored in − 80 °C freezers of Human Biomaterials Resource Centre (HBRC) of University of Birmingham. HBRC has a licence from Human Tissue Authority. FGF-23 will be tested by using Olink® Target 96 CVD II panel. BMP-10 will be tested by using the enzyme-linked immunosorbent assay (ELISA).

#### Electroanatomic mapping in the right atrium

Electroanatomic mapping of the right atrium will be performed at baseline before treatment and then restudied at the time of ablation to allow comparisons of datasets. The reason for choosing right atrial mapping over the left atrium is to reduce the risks involved in crossing the atrial septum in study participants. In our study, right atrial bipolar voltage will be measured using a high-density mapping catheter (HD-Grid, Abbott) at baseline, and bi-atrial mapping will be performed at the time of ablation. The RA mapping will be performed by an experienced electrophysiologist (M. K.).

#### Patient-reported outcome measures

The AFEQT is a validated patient-reported outcome measure designed specifically for AF-related symptoms [29]. It is a self-administered questionnaire which can be completed with 5–10 min. It is composed of 18 questions grouped into 3 functional subscales of symptoms, daily activities, and treatment concern. The score ranges from 0 (complete disability) to 100 (no disability). This will be measured at baseline, at the time of ablation, and at the end of study.

### Timeline and timing

The timeline of this feasibility study (Table 1) is designed to follow as closely as possible to the current NHS ablation pathway to minimize loss of follow-up and missing data. As the data collecting for the project is less likely to change within days, the investigators accept the window of 1 week for data collection point. This will help the researchers and the participants to reduce missing data as low as possible.

**Table 1:** Schedule of events

**Time point**	**Study period**						
	**Enrolment**	**Post enrolment**					
	**-4 weeks**	**Baseline**	**13th week**	**26th week**	**39th week**	**52nd week**	**65th week**
**Enrolment**							
**Screening**	√						
**Informed consent**	√						
**Medical history**	√						
**Kardia provision**	√						
**Intervention**							
**Liraglutide**		Daily injections					
**Assessments**							
**BMI check**		√	√				√
**Blood tests**		√	√				
**CMR**		√	√				
**Right atrial mapping**		√	√				
**AF ablation**			√				
**Review on Kardia recordings**				√	√	√	√
**AFEQT**		√	√				√

### Statistical analysis

Descriptive statistics will be used to summarize demographics and medical histories. The number of patients recruited over 52 weeks will be reported as a percentage of those eligible. The adherence to the medication will be reported as a proportion of patients who tolerate a total cumulative dose of 657 mg of liraglutide or more over 52-week follow-up period. The cumulative dose of liraglutide will be calculated for each participant, and mean and standard deviation for all participants will be reported. The percentage of change in weight from baseline to the end of follow-up will be calculated for each participant, and the proportion of participants who lost weight ≥ 5% and < 5% will be reported. 

For the secondary outcome measures, descriptive statistics according to the data type will be used to report at each predefined time point (baseline, before ablation, and end of follow-up). Paired *t*-test will be used to compare the changes for continuous variables such as changes in CMR measurements, biomarkers, and electroanatomic data to determine potential mechanistic changes in relation to weight loss. As this is a feasibility study, the analysis will be reported as point estimates and corresponding 95% confidence intervals to provide precision of estimates, and no *p*-values will be reported. A Kaplan–Meier curve will be constructed for time to recurrence of AF at 1 year following ablation. This will be performed in preparation for the calculation of sample size with appropriate power for the future clinical trial based on a time-to-event superiority analysis.

## Discussion

Most AF patients have multiple upstream risk factors, with obesity being both an independent factor and the underlying driver for many of these conditions. Weight loss and risk factor management improve outcomes from AF ablation. While currently available weight loss strategies such as intensive lifestyle modification, pharmacotherapy (for example, orlistat, naltrexone/bupropion, and phentermine/topiramate), and bariatric surgery are effective, there are limitations to availability, tolerability, and safety. Obese AF patients therefore require alternative or bridging therapy, which is speedy, safe, less invasive, and carries fewer interactions. The glucagon-like peptide 1 analogues (GLP-1As) are a new class of drug which have the potential to serve this purpose.

Effective weight loss and optimization of risk factors may reverse the AF phenotype of some patients; in some, this may mean that ablation is no longer required. Knowledge about the reversibility of the promoters of AF (such as left atrial dilatation and epicardial adiposity) is needed with respect to the proposed drug therapy. Such information before ablation will empower both patients and their treating physicians in (a) discussing expected and actual success rate, (b) better risk factor modification to improve outcome, and (c) optimal timing for ablation for better success rate for both first-time and repeat ablation. We envisage the optimized selection of patients for catheter ablation by personalizing care on the basis of biomarker profiles and cardiac imaging.

This feasibility study will determine whether our proposed intervention, liraglutide and our proposed primary end point, AF burden, or recurrence at 52 weeks after ablation are suitable for use in a large prospective randomized controlled trial. We aim to recruit both paroxysmal and persistent AF patients in this study to test the feasibility of this approach. The outcomes of recurrence are known to be different in both groups even with modification of risk factors. The specific outcome measure of recurrence between paroxysmal and persistent groups will therefore be investigated as a pre-specified sub-group analysis in the future definitive study.

### Supplementary Information

**Additional file 1.** Administrative information.

**Additional file 2.** Consent form.

## Data Availability

The dataset used for the study protocol is available from the corresponding author on reasonable request.

## Abbreviations

AF: Atrial fibrillation
AFEQT: AF Effect on QualiTy of life
AV: Atrioventricular
BMI: Body mass index
BMP: Bone morphogenic protein
CEMERGAPP: The Cardiac Electro-Mechanics Research Group Application
CMR: Cardiac MRI
cm/s: Centimetres per second
DPP-IV: Dipeptidyl peptidase 4
ECG: Electrocardiogram
ELISA: Enzyme-linked immunosorbent assay
EpAT: Epicardial adiposity
FGF: Fibroblast growth factor
GLP-1As: Glucagon-like peptide-1 analogues
GOAL-AF: Feasibility study of GLP-1 analogues for the optimization of Outcomes in obese patients undergoing AbLation for Atrial Fibrillation
HBRC: Human Biomaterials Resource Centre
HRA: Health Research Authority
mV: Millivolt
NHS: National Health Service
PIS: Patient information sheet
QEHB: Queen Elizabeth Hospital Birmingham
QoL: Quality of life
REC: Research ethics committee
SSFP: Steady-state free precession imaging
True-FISP: Siemens trade name for SSFP
UK: The United Kingdom
