# Supplementary material for: Feasibility study of Glucagon-like peptide-1 analogues for the optimization of Outcomes in obese patients undergoing AbLation for Atrial Fibrillation (GOAL-AF) protocol
Source: Pilot Feasibility Stud. 2024 Feb 21;10:36. doi: 10.1186/s40814-024-01454-y (PMC10880291; doi:10.1186/s40814-024-01454-y)
Supplement: Supplementary file 1 — Additional file 1. Administrative information. [file 40814_2024_1454_MOESM1_ESM.docx]

# Administrative information

## Protocol version number and date

Protocol Version Number -1.0 (21st July 2022)

## Sponsor

University of Birmingham, United Kingdom

## Chief investigator

Dr Manish Kalla

## Research reference number

Integrated Research Application System (IRAS) project ID-305898

Sponsor reference- RG_22-069

Research Ethics Committee (REC) approval reference- 22/WM/0213

## Trial registration

Clinical Trial Gov Identifier - NCT05221229

## Key trial contacts

| Chief Investigator | Dr Manish Kalla  1^st^ Floor Nuffield House  Queen Elizabeth Hospital  Birmingham  B15 2TH  07869147149  [Manish.Kalla@uhb.nhs.uk](mailto:Manish.Kalla@uhb.nhs.uk) |
| --- | --- |
| Trial Co-ordinator | Dr Kyaw Zaw Win  Research Office (Room 203)  2^nd^ Floor, Institute of Cardiovascular Sciences  IBR Building, College of Medical and Dental Sciences  University of Birmingham  B15 2TT  07984965157  KyawZaw.Win@uhb.nhs.uk |
| Sponsor | University of Birmingham  Edgbaston  Birmingham B15 2TT  [researchgovernance@contacts.bham.ac.uk](mailto:researchgovernance@contacts.bham.ac.uk) |
| Joint sponsor(s)/co-sponsor(s) | Not applicable |
| Funder(s) | 1. Metchley Park Medical Society and University of Birmingham Starter Fellowship 2. Abbott Investigator Initiated Study 3. BHF Accelerator Award |
| Clinical Trials Unit | Not applicable |
| Key Protocol Contributors | Prof Richard Steeds  Office 48 1^st^ Floor Nuffield House  Queen Elizabeth Hospital  Birmingham  B15 2TH  07900565937  [rick.steeds@uhb.nhs.uk](mailto:rick.steeds@uhb.nhs.uk)  Prof Jon Townend  1^st^ Floor Nuffield House  Queen Elizabeth Hospital  Birmingham  B15 2TT  John.Townend@uhb.nhs.uk  Dr Manish Kalla  1^st^ Floor Nuffield House  Queen Elizabeth Hospital  Birmingham  B15 2TH  07869147149  [Manish.Kalla@uhb.nhs.uk](mailto:Manish.Kalla@uhb.nhs.uk)  Dr Matthew Armstrong  Room 39 3^rd^ Floor Nuffield House  Queen Elizabeth Hospital  Birmingham  B15 2GW  [Matthew.Armstrong@uhb.nhs.uk](mailto:Matthew.Armstrong@uhb.nhs.uk)  Dr Kyaw Zaw Win  Research Office (Room 203)  2^nd^ Floor, Institute of Cardiovascular Sciences  IBR Building, College of Medical and Dental Sciences  University of Birmingham  B15 2TT  07984965157  [k.z.win@bham.ac.uk](mailto:k.z.win@bham.ac.uk) |
| Statistician | Samir Mehta  Medical Statistician  Birmingham Clinical Trial Unit  University of Birmingham  B15 2TT  S.MEHTA.1@bham.ac.uk |
| Committees | Not applicable |

## Source of funding

| **Funder** | **Financial and non-financial support** |
| --- | --- |
| **Metchley Park Medical Society (MPMS) Starter Fellowship**  **Contact details:**  Charlotte Maybury  Clinical Academic Training Manager  Research & Knowledge Transfer Team  Medical School Building  Vincent Drive  Edgbaston, Birmingham B15 2TT  Tel: 0121-414-6886  E-mail: c.maybury@bham.ac.uk | **Financial support**  Research Fellow Salary and consumables  **Non-financial support**  Access to University of Birmingham facilities via the Institute of Cardiovascular Sciences |
| **BHF Accelerator Award**  **Contact details:**  Caroline McKay  Research Facilitator  Research Support Services  University of Birmingham  E-mail: C.S.McKay@bham.ac.uk | **Financial support** |
| **Abbott Investigator Initiated Study**  Contact details:  Bobby Willis  Medical Affairs Manager (Abbott)  E-mail: bobby.willis@abbott.com | **Financial support** |

## Role of trial sponsor and funders

University of Birmingham will act as sole Sponsor for this project.

### Metchley Park Medical Society and University of Birmingham Starter Fellowship

The successful fellowship application requires competitive selection process including pre submission review of the protocol, review of the protocol and CVs by the review panels and the interview. The aim of the fellowship is to support the clinician to support the necessary pilot and preparatory work to apply to research funding bodies for a competitive clinical doctoral training fellowship. It does not influence the final decision regarding any of the aspects of the study.

### BHF Accelerator Award

BHF Accelerator Award is a peer-reviewed pump priming grant. Dr Kalla has been awarded for AF biomarkers work in collaboration with the AF biomarker research group. This grant does not influence the final decision regarding any of the aspects of the study.

### Abbott Investigator Initiated Study

Dr Kalla has secured the funding for electroanatomic mapping and consumables of the study. Abbott does not have influence on the final decision regarding any of the aspects of the study.

# Methods

### Withdrawal criteria

Serious adverse events (SAE) are not expected in the study due to the study inclusion/exclusion criteria for the participants. All study procedures, including use of liraglutide, are part of standard medical care. If Suspected Unexpected Serious Adverse Reaction (SUSAR) occurs in the study, immediate discussion with the chief investigator and the team will take place to consider withdrawal of the participant and stopping of the study.

If a participant who has provided informed consent wanted to withdraw or lost capacity to consent during the study, he or she will be withdrawn from the study. Identifiable data or blood samples that have already been collected with consent will be kept and used in the study. No further data or blood samples would be collected, nor would any other research procedures be performed on or in relation to the participant.

### Data collection and source document identification

The source documents and data are from QEHB patient clinical records, lab records, MRI reporting system and ablation reporting system. These will be stored according to QEHB clinical standards. The source documents for AF biomarkers are the reports from collaborators. The original reports will be stored as source documents.

The Case Report Form (CRF) is created to capture data required for the study. It will capture age, gender, ethnicity, medical history, list of current medications, height, weight, BMI, lab results (Full Blood Count, Liver Function, Kidney Function, Lipid Profile, AF Biomarkers), heart MRI data (Ejection Fractions of left ventricle and left atrium, left atrial strain, left atrial epicardial adiposity, left atrial scar percentage, cumulative does of liraglutide, AFEQT scores, episodes of AF recurrence on Kardia.

### Data storage

All recruited patients will be assigned a study number. Passwords and encryption will be used to secure the data whenever possible and for appropriate storage of research samples. Baseline tests are part of standard of care and therefore the research team will require linked study and clinical identifiers to compile a full data set. These data will be stored with encryption according to NIHR data protection guidance.

### Data monitoring and auditing

The study will be monitored and/or audited by University of Birmingham under their remit as Sponsor and other regulatory bodies to ensure adherence to Good Clinical Practice and the UK Health Policy Framework for Health and Social Care.

### Declaration of interests

The study team members have nothing to declare in financial and other competing interests.

### Post study care and indemnity

All the participants will be followed up as per NHS clinical standard in QEHB. University of Birmingham acts as the Sponsor to this study. Delegated responsibilities will be assigned to the Chief Investigator and QEHB taking part in this study. The non-commercial OID will be used with all participating sites detailing their local responsibilities. QEHB holds standard NHS Hospital indemnity and insurance cover with NHS Litigation Authority for NHS Trusts in England, which apply to this study.

### Access to the final dataset and dissemination policy

The study data set will only be available to the research team. The research team holds the ownership of the data following completion of the study. Then the data will be analysed and tabulated, and the final study report will be prepared. The participating investigators will have rights to publish the data with permission from the chief and co-chief investigators. All the funding and supporting bodies will be acknowledged in all published papers of the study. However, they do not hold the publication rights of the data from the study.

### Reporting adverse events

All SAEs occurring from the time of written informed consent until the last day of follow up of the last participant will be recorded on the SAE Form and email to the Sponsor within 24 hours of the research staff becoming aware of the event. Once all resulting queries have been resolved, the Sponsor will request the original form should also be posted to the Sponsor and a copy to be retained on site.

### Adherence to the protocol

Adherence to the protocol is very important in producing high quality output. However, accidental deviation from it can occur in any project. To mitigate this, the weekly research meetings with allocated time have been arranged to discuss any issue raised by the team members. This is to further prevent recurrent deviations which can potentially lead to a serious breach.

### Amendments

The process of amendments will follow strictly according to HRA NHS organisation guidelines. The research team and the sponsor are responsible to decide for amendment of the protocol, either substantially or non-substantially. If substantial changes are required, these changes will be informed to REC, and R&D QEHB. All the changes and amendment on protocol and related documents will be tracked and versioned.
