# Supplementary material for: Feasibility study of Glucagon-like peptide-1 analogues for the optimization of Outcomes in obese patients undergoing AbLation for Atrial Fibrillation (GOAL-AF) protocol
Source: Pilot Feasibility Stud. 2024 Feb 21;10:36. doi: 10.1186/s40814-024-01454-y (PMC10880291; doi:10.1186/s40814-024-01454-y)
Supplement: Supplementary file 2 — Additional file 2. Consent form. [file 40814_2024_1454_MOESM2_ESM.docx]

University Hospital Birmingham  
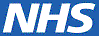


Queen Elizabeth Hospital

Edgbaston

Birmingham  B15 2TH

**Direct Line Tel: 0121 3714042**

**Fax: 0121 3714044**

##### CONSENT FORM (Patient)

**Title of Project:**

**GOAL-AF (Feasibility study of GLP-1 analogues for the optimization of Outcomes in high BMI patients undergoing AbLation for Atrial Fibrillation)**

**Name of Researchers:**

Dr Kyaw Zaw Win (Research Fellow), Prof Richard Steeds (Consultant Cardiologist), Dr Manish Kalla (Consultant Cardiologist), Dr Matthew Armstrong (Consultant Liver Transplant Physician)

Please initial box

1. I confirm that I have read and understand the information sheet dated () (version) for the above study.
2. I understand that my participation is voluntary and that I am free to withdraw at any time without my medical care or legal rights being affected.
3. I understand that relevant data collected during the study may be looked at by individuals from the

University of Birmingham, from regulatory authorities or from the NHS Trust, where it is relevant

to my taking part in this research. I give permission for these individuals to have access to my records.

1. I understand that information held and managed by central UK NHS bodies and NHS Trusts may be

used in order to provide information about my health status during and after the study.

1. I agree to my data/tissue being used for future ethically approved studies

Yes

No

1. I agree to my General Practitioner being informed of my participation in the study

Yes

No

1. I agree to take part in this study

| Name of patient | Date | Signature |
| --- | --- | --- |
| Name of person receiving consent | Date | Signature |
| Researcher | Date | Signature |

The signed consent form will be scanned and imported onto the patient information record system (PICS) of QEHB. The hard copy of the consent form will be stored in secured cabinets during the study and archived according to the University of Birmingham archiving policy. A copy will be given to you.
